# Financial well‐being as a mediator of the relationship between multimorbidity and health‐related quality of life in people with cancer

**DOI:** 10.1002/cam4.6204

**Published:** 2023-06-07

**Authors:** Winnie K. W. So, Doreen W. H. Au, Dorothy N. S. Chan, Marques S. N. Ng, Kai Chow Choi, Weijie Xing, Mandy Chan, Suzanne S. S. Mak, Pui Shan Ho, Man Tong, Cecilia Au, Wai Man Ling, Maggie Chan, Raymond J. Chan

**Affiliations:** ^1^ The Nethersole School of Nursing, Faculty of Medicine The Chinese University of Hong Kong Hong Kong China; ^2^ School of Nursing and Health Studies Hong Kong Metropolitan University Hong Kong China; ^3^ School of Nursing Fudan University Shanghai China; ^4^ Department of Clinical Oncology Prince of Wales Hospital Hong Kong China; ^5^ Department of Clinical Oncology Tuen Mun Hospital Hong Kong China; ^6^ Department of Clinical Oncology Pamela Youde Nethersole Eastern Hospital Hong Kong China; ^7^ Tung Wah Eastern Hospital Hong Kong China; ^8^ Caring Futures Institute, College of Nursing and Health Sciences Flinders University Adelaide Australia

**Keywords:** cancer patients, financial well‐being, multimorbidity, quality of life

## Abstract

**Background:**

It is unknown whether financial well‐being mediates the impact of multimorbidity on the health‐related quality of life (HRQoL) of cancer patients.

**Methods:**

Participants were recruited from three outpatient oncology clinics of Hong Kong public hospitals. Multimorbidity was assessed using the Charlson Comorbidity Index. Financial well‐being, the mediator of the association between multimorbidity and HRQoL outcomes, was assessed using the Comprehensive Score for Financial Toxicity Functional Assessment of Chronic Illness Therapy. The HRQoL outcomes were assessed using the Functional Assessment of Cancer Therapy – General (FACT‐G) and its four sub‐dimensions. Mediation analyses were conducted using SPSS PROCESS v4.1.

**Results:**

Six‐hundred and forty cancer patients participated in the study. Multimorbidity had a direct effect on FACT‐G scores independent of financial well‐being (β for path c’ = −0.752, *p* < 0.001). In addition, multimorbidity had an indirect effect on FACT‐G scores through its effect on financial well‐being (β for path a = −0.517, *p* < 0.05; β for path b = 0.785, *p* < 0.001). Even after adjustments were made for the covariates, the indirect effect of multimorbidity on FACT‐G via financial well‐being remained significant, accounting for 38.0% of the overall effect, indicating partial mediation. Although there were no statistically significant associations between multimorbidity, social well‐being, and emotional well‐being, the indirect effects of multimorbidity on physical and functional well‐being through financial well‐being remained significant.

**Conclusions:**

Poor financial well‐being attributable to multimorbidity partially mediates the direct impact of chronic conditions on HRQoL in Chinese cancer patients, particularly their physical and functional well‐being.

## Graphical abstract

The study's findings emphasize the significance of financial health when managing and treating multiple chronic conditions in cancer patients. Future healthcare policies pertaining to health‐related quality of life for cancer populations should thus prioritize financial health.

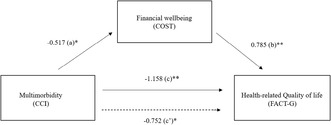

## 1 INTRODUCTION

Cancer is more likely to coexist with two or more chronic illnesses as people age, and the occurrence of multimorbidity has a detrimental impact on health service utilization and cost, as well as the risk of early mortality.^1^, ^2^ Recent studies have also demonstrated that multimorbidity has significant negative impacts on health‐related quality of life (HRQoL), a multidimensional concept that takes into account an individual's physical, psychological, and social functioning over time.^3^, ^4^ These studies have shown that a cumulative multimorbidity burden is associated with low HRQoL, regardless of the chronic conditions involved. For instance, compared with older cancer survivors reporting only one comorbidity, those with two or more comorbid conditions reported worse pain, fatigue, and greater physical function limitations. However, although multimorbidity is one of the most common health problems, it has received little attention in public health agendas.

Early initiatives and research were entirely focused on treatments and improving the outcomes of the large proportion of patients with cancer who have pre‐existing chronic conditions with no concern of the patients' financial situation.^5^ However, with the rising cost of cancer treatments, the adverse impact of increased co‐payment costs and the financial difficulties faced by cancer patients have drawn considerable attention to cancer patients' financial health/well‐being.^6^ This adverse effect has been denoted “financial hardship,” “financial burden,” “financial distress,” and, more recently, “financial toxicity,” which encompasses both the subjective financial distress and the objective financial burden experienced by patients as a result of their cancer diagnosis and treatment.^7^, ^8^, ^9^, ^10^, ^11^, ^12^ Regardless of the term used, there has been exponential growth in the literature on the detrimental impact of financial toxicity on the HRQoL of cancer patients.^6^, ^13^, ^14^ Cancer patients experience financial toxicity regardless of their type of health system (user‐pay, universal, or hybrid). Moreover, the clinical relevance of financial toxicity has been demonstrated to be equivalent to that of physical and psychological distress, which can affect multiple aspects of life and, ultimately, overall HRQoL.^15^ Comparing cancer patients with higher levels of financial toxicity to those with lower levels of financial toxicity, it has been found that the former have lower overall HRQoL.^11^

The National Health Interview Survey (2015–2017) found that 137.1 million Americans had financial toxicity episodes. Financial toxicity was higher in those with more chronic illnesses than in those with fewer chronic illnesses.^16^ This finding was consistent with the findings of Jones and colleagues, who reported that comorbidity increased the financial burden for cancer patients despite any buffering provided by medical insurance coverage (e.g., Medicare).^17^ While financial toxicity may be attributable to the cost of cancer treatment, the financial burden that arises from multimorbidity conditions may severely decrease cancer patients' HRQoL. However, this area has rarely been investigated.^18^

Although studies have examined the relationships between multimorbidity, financial well‐being, and HRQoL, there is limited research on how cancer patients' financial well‐being mediates the relationship between multimorbidity and HRQoL. Identification of this mediating role may help the healthcare system and providers to address the financial concerns of cancer patients who are managing multiple chronic conditions. Thus, the aim of this study was to obtain new information on how financial well‐being may improve the HRQoL of cancer populations with multimorbidity.

## 2 METHOD

### 2.1 Participants, settings, and data collection

A total of 640 cancer patients were recruited through convenience sampling from three outpatient oncology clinics of Hong Kong public hospitals from November 2018 to January 2019. Under Hong Kong's universal healthcare system, 90% of healthcare costs are covered by tax revenue, but patients may need to pay out‐of‐pocket for non‐formulary drugs, such as expensive targeted therapy and immunotherapy drugs. Given the limited uptake of health insurance, financial well‐being remains a practical challenge in cancer care.^19^, ^20^

Patients aged 18 and over with any cancer type who were awaiting follow‐up were invited to participate in this study. The research team approached potential participants during their routine medical appointments. After eligible patients had provided informed consent, they were invited to complete a demographic questionnaire that covered the study's outcomes. The questionnaires were immediately returned to the researcher upon completion.

### 2.2 Instruments

The Functional Assessment of Cancer Therapy – General (FACT‐G) was used to measure HRQoL.^21^, ^22^ The FACT‐G, which was initially created in English, has been translated into traditional Chinese and validated among a group of Chinese cancer patients.^23^, ^24^, ^25^ This 27‐item assessment has subscales for physical well‐being (PWB), social/family well‐being (SWB), emotional well‐being (EWB), and functional well‐being (FWB). Items are scored on a 5‐point rating scale, where 0 indicates “not at all,” 1 “a little bit,” 2 “somewhat,” 3 “quite a bit,” and 4 “very much.” Reverse scoring was used for negative items. A better HRQoL is indicated by higher subscale and overall scores.^26^

Multimorbidity was measured using the Charlson Comorbidity Index (CCI). The CCI is based on a list of 19 chronic health conditions, which were identified from the participants' self‐reports that were then verified by our research team from the participants' medical charts. Each condition is given a weight between 1 and 6, and the index score is the sum of the weights for all of the identified conditions. An index score of 0 indicates no comorbid conditions, and a higher score indicates a higher level of comorbidity. The CCI has excellent interrater reliability, with extremely high agreement between the self‐reports and medical charts.^27^ The CCI has been used in several Chinese cancer patient settings^28^, ^29^ and was shown to be clinically useful not only in providing a valid assessment of a patient's unique clinical situation but also in distinguishing major diagnostic and prognostic differences in subgroups of patients with the same medical diagnosis.^30^

The Comprehensive Score for Financial Toxicity Functional Assessment of Chronic Illness Therapy (COST) was used to assess the participants' financial well‐being.^31^ Each item is rated on a 5‐point scale (0 = “not at all,” 1 = “a little bit,” 2 = “somewhat,” 3 = “quite a bit,” and 4 = “very much”). The COST score is computed by adding the scores of the 11 items (items 2, 3, 4, 5, 8, 9 and 10 were reverse‐scored). The Chinese version of the COST was validated in this sample, with a higher score indicating greater financial well‐being.^7^

### 2.3 Data analysis

Univariate analyses were conducted to describe the characteristics of the sample in its totality. The normality of variables with continuous data, including the total and subscale scores of FACT‐G, CCI, and COST, was assessed based on their skewness statistics and normal probability plots. Cronbach's alpha coefficients were calculated to verify the internal consistency of FACT‐G and COST, with an alpha >0.70 considered acceptable.^32^

**FIGURE 1:**
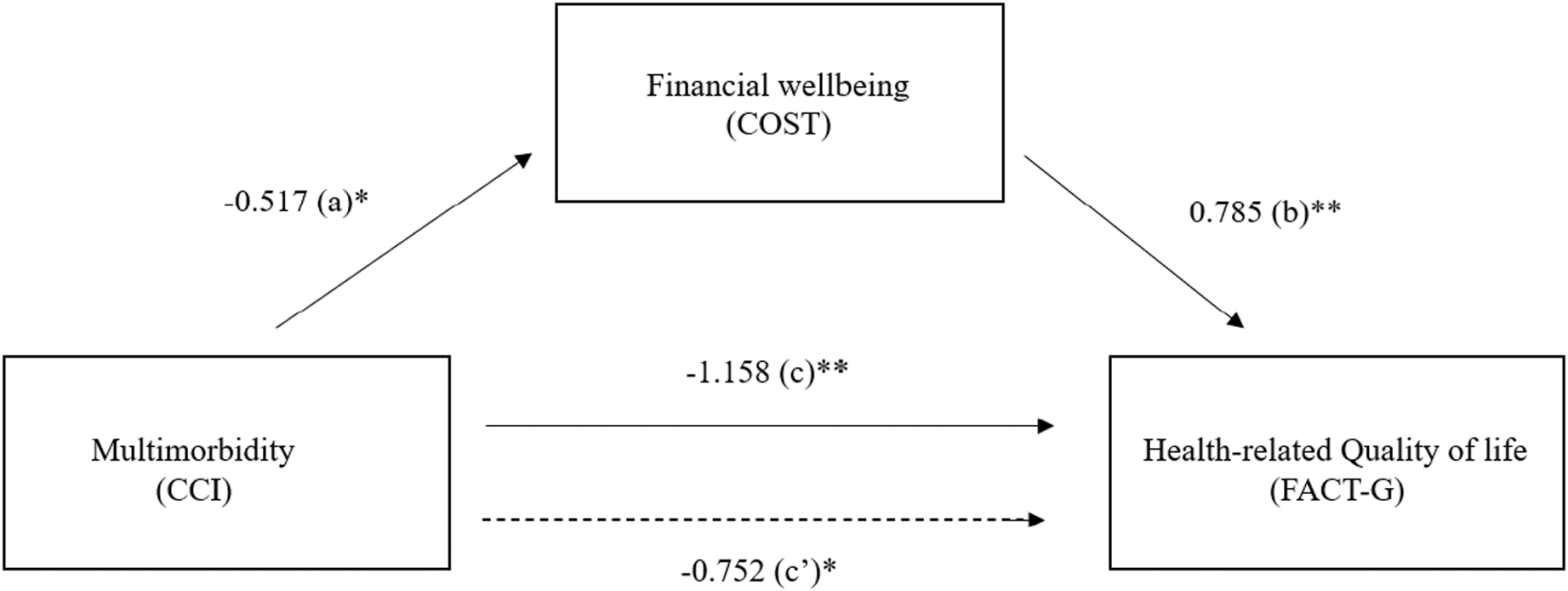
A mediation analysis for the relationship of multimorbidity and financial wellbeing with health‐related quality of life. *p < 0.05; **p < 0.01. CCI, Charlson Comorbidity Index; COST, The COmprehensive Score for financial Toxicity; FACT‐G, The Functional Assessment of Cancer Therapy‐General. In the mediation model, the indirect effect is the product of path coefficients a (Multimorbidity →Financial wellbeing) and b (Financial wellbeing → FACT‐G). The direct effect is the coefficient c’ (multimorbidity → FACT‐G). The total effect (c) is equal to the sum of the direct and indirect (c’ + ab).

The mediating effect of financial well‐being on the association between multimorbidity and FACT‐G was tested based on the mediation paths devised by Baron and Kenny, as illustrated in Figure 1.^33^ Single‐variable mediation analyses were conducted using PROCESS v4.1 model #4 (single mediator), and the coefficients were calculated using a bootstrap estimation approach with 10,000 samples.^34^ Multimorbidity determined by CCI was the predictor variable, financial well‐being determined by COST was the mediator, and the outcome variables were the overall FACT‐G score and its sub‐dimensions PWB, SWB, EWB, and FWB.

The mediating effect of financial well‐being on the association between multimorbidity and HRQoL was examined using the approach devised by Baron and Kenny, as illustrated in Figure 1.^35^ Multimorbidity determined by CCI was the explanatory variable, financial well‐being determined by COST was the mediator, and the outcome variables were the overall FACT‐G score and its sub‐dimensions PWB, SWB, EWB, and FWB. Linear regression analyses were conducted using the SPSS macro PROCESS v4.1 (model #4). The multicollinearity of the independent variables in the linear regressions was assessed using their variance inflation factors (VIFs). The significance of the mediating effect on a HRQoL outcome was assessed based on the bias‐corrected bootstrapped 95% confidence interval of the indirect effect of multimorbidity on the underlying outcome through financial well‐being, with 10,000 replications.^36^ Further analyses with adjustments for covariates, such as age, sex, marital status, educational attainment, household monthly income, employment status, living arrangements, and insurance status, were conducted to examine the robustness of the mediation analysis results.

The online calculator created by Schoemann and his colleagues was used to perform a power analysis using a Monte Carlo simulation for indirect effects with the default settings (i.e., 1000 replications, 20,000 Monte Carlo draws per replication, random seed 1234, 95% CI).^37^ All of the statistical analyses were two‐sided with a significance level of 0.05 and were conducted using SPSS (version 26).

## 3 RESULTS

### 3.1 Characteristics of the study population

The majority (64.2%) of the 640 participants in this study were female, and the participants had an average age of 59.9 (SD 11.1) years. Most of the participants (72.0%) were married, lived with others (91.4%), and had a secondary school education (50.8%). Approximately 40% had monthly household incomes of less than 10,000 HKD (approximately 1272 USD), 66.3% lacked health insurance, and a large majority (79.2%) were unemployed. Breast cancer (36.4%) was the most common diagnosis. The demographic characteristics of the participants are presented in Table 1.

**TABLE 1:** Demographics and cancer diagnosis of participants (*n* = 640).

Characteristics	*n*	%
Gender		
Male	229	35.8%
Female	411	64.2%
Marital status		
Single/divorced/widowed	179	28.0%
Married/cohabitation	461	72.0%
Education		
No formal education/primary	202	31.6%
Secondary	325	50.8%
Post‐secondary or above	113	17.7%
Living alone		
Yes	55	8.6%
No	585	91.4%
Household monthly income (HKD)		
<10,000	259	40.5%
10,000–29,999	197	30.8%
≥30,000	144	22.5%
Do not know	40	6.3%
Employment status		
Employed	133	20.8%
No employment	507	79.2%
Specific sites of the cancer		
Breast	233	36.4%
Gastric and colorectal	162	25.3%
Genitourinary	35	5.5%
Gynecological	27	4.2%
Head and neck	34	5.3%
Hematological	25	3.9%
Lung	104	16.3%
Others	20	3.1%
Have medical insurance		
Yes	216	33.8%
No	424	66.3%

	Mean	SD
Age (years)	59.9	11.1

### 3.2 Correlations between CCI, COST, and FACT‐G

Table 2 shows that the mean score of CCI was 5.48 (SD = 1.97), that of COST was 20.1 (SD = 8.8), and that of FACT‐G was 71.6 (SD = 15.5). The distribution of scores and the number of comorbidities did not deviate from normality, with skewness ranging from −0.671 to 0.132. The Cronbach's alpha value for COST was 0.86 and that for FACT‐G was 0.90, indicating good internal consistency. Bivariate correlations revealed that multimorbidity was negatively correlated with financial well‐being, as well as with overall quality of life and each of its subscales. However, the correlations between multimorbidity and the SWB subscale and between multimorbidity and the EWB subscale were not significant.

**TABLE 2:** Correlation Matrix, means, and standards deviations for FACT‐G and subscales, COST, and CCI.

	FACT‐G	PWB	SWB	EWB	FWB	COST	CCI
Score range	0–108	0–28	0–28	0–24	0–28	0–44	0–12
Mean	71.6	19.2	19.1	17.7	15.7	20.1	5.5
SD	15.5	5.8	5.1	4.4	5.4	8.8	2.0
FACT‐G	–	0.81**	0.66**	0.73**	0.79**	0.47**	−0.141**
PWB	–	–	0.32**	0.51**	0.54**	0.34**	−0.130**
SWB	–	–	–	0.30**	0.35**	0.23**	−0.033
EWB	–	–	–	–	0.46**	0.42**	−0.032
FWB	–	–	–	–	–	0.39**	−0.209**
COST	–	–	–	–	–	–	−0.094*

*Note*: *denoted as *p*‐value <0.05; **denoted as *p*‐value <0.01.

Abbreviations: CCI, Charlson Comorbidity Index; COST, The COmprehensive Score for financial Toxicity; EWB, emotional wellbeing; FACT‐G, The Functional Assessment of Cancer Therapy‐General; FWB, functional wellbeing; PWB, Physical wellbeing; SD, standard deviations; SWB, Social/family wellbeing.

### 3.3 Mediation analysis

All of the VIF values were close to 1, indicating that multicollinearity was not a problem in any of the regression models. As shown in Table 3 and Figure 1, the mediating effect of financial well‐being on the association between multimorbidity and the total FACT‐G score was examined using the PROCESS macro with a bootstrapping procedure. The mediation analysis showed that multimorbidity had a direct effect on FACT‐G independent of financial well‐being (unstandardized regression coefficient, β for path c’ = −0.752, *p* < 0.001). In addition, multimorbidity had an indirect effect on FACT‐G through its effect on financial well‐being. Multimorbidity was negatively associated with financial well‐being (β for path a = −0.517, *p* < 0.05), and financial well‐being was positively associated with FACT‐G (β for path b = 0.785, *p* < 0.001). Moreover, multimorbidity and financial well‐being were significantly associated with FACT‐G even after all of the covariates in Model 2 were adjusted (β for path c’ = −0.644, *p* < 0.05; β for path a = −0.503, *p* < 0.05; β for path b = 0.784, *p* < 0.001). The bootstrapping showed that the indirect effect of multimorbidity on FACT‐G via financial well‐being remained significant (the 95% CI did not cover 0) even after adjustments were made to all of the covariates, explaining 38.0% the total effect (partial mediation). Monte Carlo power analysis was able to detect an indirect effect using about 70% power.

**TABLE 3:** A mediating analysis of financial wellbeing for the relationship between multimorbidity and health‐related quality of life.

Path	Model 1		Model 2	
	β	95% CI	β	95% CI
Multimorbidity → Financial wellbeing, a	−0.517*	−0.870 to −0.163	−0.503*	−0.856 to −0.151
Financial wellbeing → FACT‐G, b	0.785**	0.663 to 0.907	0.784**	0.658 to 0.909
Total effect, c	−1.158**	−1.778 to −0.537	−1.038*	−1.668 to −0.409
Direct effect, c’	−0.752*	−1.311 to −0.193	−0.644*	−1.213 to −0.075
Indirect effect, ab	−0.405*	−0.699 to −0.127	−0.394*	−0.691 to −0.104
Ratio of indirect to total effect mediated (%)	35.0%		38.0%	

*Note*: Model 1 Unadjusted. Model 2 adjusted for age, gender, income, marital status, education attainment, family income, living arrangement, employment status, and insurance status. Number of bootstrap replications for bias‐corrected bootstrap confidence intervals: 10,000. *p < 0.05, **p < 0.001.

In the mediation model, the indirect effect is the product of path coefficients a (Multimorbidity →Financial wellbeing) and b (Financial wellbeing → FACT‐G). The direct effect is the coefficient c’(multimorbidity → FACT‐G). The total effect (c) is equal to the sum of the direct and indirect (c’ + ab).

Abbreviations: CI, confidence interval; FACT‐G, The Functional Assessment of Cancer Therapy‐General.

Table 4 demonstrates the mediating effect of financial well‐being on the association between multimorbidity and the FACT‐G subscales. The mediation analysis showed that multimorbidity had direct effects on PWB and FWB independent of financial well‐being (β for path c’ = −0.279, *p* < 0.05; β for path c’ = −0.471, *p* < 0.001, respectively). Furthermore, multimorbidity had indirect effects on PWB and FWB through its effect on financial well‐being. Multimorbidity was negatively associated with financial well‐being (β for path a = −0.517, *p* < 0.05), and financial well‐being was positively associated with PWB (β for path b = 0.215, *p* < 0.001) and FWB (β for path b = 0.228, *p* < 0.001). In addition, multimorbidity and financial well‐being remained significantly associated with PWB and FWB even after adjustments were made for all of the covariates in Model 2 (β for path c’ = −0.245, *p* < 0.05; β for a = −0.503, *p* < 0.05; β for path b = 0.214, *p* < 0.001; β for path c’ = −0.363, *p* < 0.001; β for a = −0.503, *p* < 0.05; β for path b = 0.233, *p* < 0.001, respectively). Bootstrapping revealed that the indirect effects of multimorbidity on PWB and FWB via financial well‐being remained significant even when all of the covariates were controlled, accounting for 30.5% of the total effect (partial mediation) for PWB (power of 0.69) and 24.4% of the total effect for FWB (power of 0.70).

**TABLE 4:** A mediating analysis of financial wellbeing for the relationship between multimorbidity and dimensions of health‐related quality of life.

Path	Model 1		Model 2	
	β	95% CI	β	95% CI
Multimorbidity →Financial wellbeing, a	−0.517*	−0.870 to −0.163	−0.503**	−0.856 to −0.151
Financial wellbeing →**PWB**, b	0.215**	0.166 to 0.263	0.214**	0.163 to 0.264
Total effect, c	−0.390*	−0.623 to −0.157	−0.353*	−0.592 to −0.114
Direct effect, c’	−0.279*	−0.501 to −0.057	−0.245*	−0.474 to −0.017
Indirect effect, ab	−0.111*	−0.196 to −0.034	−0.108*	−0.193 to −0.028
Ratio of indirect to total effect mediated (%)	28.4%		30.5%	
Financial wellbeing → **SWB**, b	0.132**	0.088 to 0.176	–	–
Total effect, c	N.S.			
Direct effect, c’	N.S.			
Indirect effect, ab	N.S.			
Ratio of indirect to total effect mediated (%)	N.S.			
Financial wellbeing → **EWB**, b	0.211**	0.175 to 0.25	–	–
Total effect, c	N.S.			
Direct effect, c’	N.S.			
Indirect effect, ab	N.S.			
Ratio of indirect to total effect mediated (%)	N.S.			
Financial wellbeing → **FWB**, b	0.228**	0.184 to 0.271	0.233**	−0.562 to −0.164
Total effect, c	−0.589**	−0.801 to −0.376	−0.480**	−0.694 to −0.266
Direct effect, c’	−0.471**	−0.669 to −0.272	−0.363**	−0.562 to −0.164
Indirect effect, ab	−0.118*	−0.204 to −0.038	−0.117*	−0.206 to −0.031
Ratio of indirect to total effect mediated (%)	20.0%		24.4%	

*Note*: Model 1 Unadjusted. Model 2 adjusted for age, gender, income, marital status, education attainment, family income, living arrangement, employment status, and insurance status. Number of bootstrap replications for bias‐corrected bootstrap confidence intervals: 10,000. *p < 0.05, **p < 0.001.

Abbreviations: CI, confidence interval; EWB, Emotional wellbeing; FACT‐G, The Functional Assessment of Cancer Therapy‐General; FWB, Functional wellbeing; N.S., Non significant; PWB, Physical wellbeing; SWB, Social/family wellbeing.

## 4 DISCUSSION

This study is the first to examine the potential role of financial well‐being in mediating the relationship between multimorbidity and HRQoL in cancer patients. Our findings suggest that financial well‐being partially mediates the relationship between multimorbidity and HRQoL in Chinese cancer patients. The findings also imply that poor financial well‐being induced by multimorbidity can affect HRQoL in cancer patients, particularly in terms of physical and functional well‐being. A better understanding of these complex relationships will inform the development of interventions for optimizing the management of multimorbidity and financial well‐being, that is, interventions that focus on more than cancer treatment in populations of cancer patients.

The data in this study are consistent with the well‐established negative correlations between multimorbidity, financial well‐being, and overall HRQoL.^38^ This study further explored the effects on the specific aspects of HRQoL and found no statistically significant associations between multimorbidity and social or emotional well‐being. These results are consistent with a previous study finding no obvious associations between multimorbidity and mental health for patients with the four most common cancers (i.e., breast, colorectal, lung, and prostate cancer).^35^ It was also found that, in rare cancers, the clinical severity of the tumor, such as the presence or absence of metastases or comorbidities, and the tumor heterogeneity, such as the primary site, had obvious impact on physical symptoms and financial problems.^36^, ^39^ These studies highlighted that cancer patients may experience a range of competing comorbidities that are significant to HRQoL and, in some cases, have a greater impact on physical health than mental health.

This study contributes to the evidence on multimorbidity and financial well‐being as predictors of HRQoL in cancer patients by conducting a mediation analysis in which the total effect is divided into direct and indirect effects. This is the first study to show that poor financial well‐being attributable to multimorbidity partially mediates the direct impact of the chronic conditions on HRQoL in Chinese cancer patients. Our results support the finding of an earlier study that financial hardship caused by treatment adherence was detrimental to the overall well‐being of people with chronic conditions, particularly with regard to physical and functional well‐being.^18^ That study discovered that because of financial hardship, participants had trouble filling prescriptions, getting the care they needed, or getting the medications that doctors prescribed. Such experiences had a negative impact on the participants' physical well‐being, causing them to struggle with fatigue, feel ill or ill at ease, be unable to meet their family's needs, be bothered by the side effects of treatment, and spend more time in bed than they had previously. In addition, financial burden was negatively associated with functional well‐being, which means that compared with people with chronic conditions who do not have poor financial well‐being, people with chronic conditions who have poor financial well‐being are less able to work, enjoy life, or engage in their usual leisure activities. They may also find it difficult to accept their illnesses, experience sleep problems, or be dissatisfied with their current HRQoL.^12^

Our findings should be interpreted with caution because of the following study limitations. First, the survey was cross‐sectional, which limits our ability to predict the causal pathway between multimorbidity, financial well‐being, and HRQoL. However, we used multicollinearity diagnostic statistics in this study to ensure unbiased coefficient estimates from the cross‐sectional dataset. Furthermore, the mediation analysis conducted in this study did not differentiate between cancer types, making it impossible to assess the relative impact of poor financial well‐being on different cancer types. As various cancer types and chronic conditions may have varying levels of associations with both physical and mental health, future research could use a much larger data set to separate the effects of comorbidities and financial well‐being on the health status of people with different types of cancer.^40^ Additionally, even though the 19 comorbid conditions in the CCI represent the majority of comorbidities experienced by cancer patients, the exclusion of other chronic conditions may have resulted in an underestimation of the impact on the sub‐dimensions of the HRQoL.

Together with the findings of previous studies, the results of this study offer the insight that consideration of various potential risk and protective factors for health outcomes, including financial well‐being, is a necessary step for identifying people with cancer who may need frequent monitoring and assistance with financial counseling and chronic disease management as they transition from treatment to survivorship.^4^ This implication is important because many interventions to address financial toxicity focus on the direct and indirect costs of cancer and its treatment, which are usually delivered by oncology social workers and cancer‐specific not‐for‐profit organizations.^41^, ^42^ First, financial navigation and counseling interventions continue to be developed in oncology, and it is important that such interventions also address financial concerns related to conditions other than cancer. Second, healthcare policy makers should consider taking a more holistic approach than they currently take to enhance support for addressing the financial concerns of people with multimorbidity. The current study's findings on the relationships between multimorbidity, financial well‐being, and HRQoL emphasize the significance of financial health when managing and treating multiple chronic conditions in cancer patients. Future healthcare policies pertaining to HRQoL for cancer populations should thus prioritize financial health. In addition, despite the fact that there were no statistically significant gender differences in HRQoL in our study population, it may be worthwhile to increase the sample size in the near future to see if subgroup differences exist in the study relationship.

## AUTHOR CONTRIBUTIONS

**Winnie K. W. So:** Conceptualization (equal); funding acquisition (lead); methodology (lead); project administration (lead); supervision (lead); writing – review and editing (equal). **Doreen W. H. Au:** Conceptualization (lead); data curation (lead); formal analysis (lead); writing – original draft (equal). **Dorothy N. S. Chan:** Writing – review and editing (equal). **Marques S. N. Ng:** Writing – review and editing (equal). **Kai Chow Choi:** Data curation (supporting); formal analysis (supporting); writing – review and editing (equal). **Weijie Xing:** Writing – review and editing (equal). **Mandy Chan:** Writing – review and editing (equal). **Suzanne S. S. Mak:** Writing – review and editing (equal). **Pui Shan Ho:** Writing – review and editing (equal). **Man Tong:** Writing – review and editing (equal). **Cecilia Au:** Writing – review and editing (equal). **Wai Man Ling:** Writing – review and editing (equal). **Maggie Chan:** Writing – review and editing (equal). **Raymond J. Chan:** Writing – review and editing (equal).

## FUNDING INFORMATION

This study was funded by The Chinese University of Hong Kong (reference number: CA‐1806). The funding institution provided financial support for the research. The funding institution had no role in the study design, the data collection and analysis, the decision to publish or the preparation of the manuscript. RJC receives salary support from the Australian National Health and Medical Research Council (APP1194051).

## CONFLICT OF INTEREST STATEMENT

The authors declare no conflict of interest.

## ETHICAL APPROVAL

Ethics approval was obtained from the Joint CUHK‐NTEC Clinical Research Ethics Committee (reference number: 2018.456). All procedures performed were in accordance with the ethical standards of the Joint CUHK‐NTEC Clinical Research Ethics Committee and the institutional research boards of the study sites.

## PATIENT CONSENT STATEMENT

Informed consent was obtained from all participants included in the study.

## Data Availability

The data that support the findings of this study are available upon request, but restrictions apply to the availability of these data.
